# Examining the Utility of the Early Childhood Development Index (ECDI) among Children in the Nigeria Context

**DOI:** 10.3390/children11030361

**Published:** 2024-03-19

**Authors:** Ngozi V. Enelamah, Smitha Rao, Margaret Lombe, Mansoo Yu, Chrisann Newransky, Melissa L. Villodas, Andrew Foell, Von Nebbitt

**Affiliations:** 1 Department of Social Work, University of New Hampshire, Durham, NH 03824, USA; ngozi.enelamah@unh.edu; 2 College of Social Work, The Ohio State University, Columbus, OH 43210, USA; rao.506@osu.edu; 3 School of Social Work, Boston University, Boston, MA 02215, USA; lombem@bu.edu; 4 College of Health Sciences, University of Missouri, Columbia, MO 65211, USA; 5 School of Social Work, Adelphi University, Garden City, NY 11530, USA; cnewransky@adelphi.edu; 6 Department of Social Work, George Mason University, Fairfax, VA 22030, USA; mvilloda@gmu.edu; 7 College of Social Work, University of Illinois Chicago, Chicago, IL 60607, USA; afoell@uic.edu; 8 School of Social Work, Morgan State University, Baltimore, MD 21251, USA; von.nebbitt@morgan.edu

**Keywords:** early childhood development, children, culturally appropriate measures, low- and middle-income countries, item response theory

## Abstract

An estimated 6 million children under the age of five in Nigeria (out of nearly 31 million) risk not reaching their full developmental potential. The dearth of context-relevant measures poses a challenge to the planning and implementation of effective interventions. This study assesses the utility of the Early Childhood Development Index (ECDI) in Nigeria. We used the Multiple Indicator Cluster Surveys to track progress among 3- to 4-year-old children (n = 11,073); 3-year-old, 51%; female, 49%. Using random calibration samples, the results from psychometric tests indicate that while over half of the children were on track in their development based on the ECDI, the instrument had low to average internal consistency and weak face validity, suggesting an inadequacy in capturing ECD information of value. At the outset of the launch of the new ECDI2030, the results of this study point to the need for continued discourse and advocacy for the use of culturally appropriate measures of child development, and a child-centered community engagement approach. This is essential in ensuring accountability and responsive interventions for the children served and their families.

## 1. Introduction

Early childhood is associated with the rapid development of physical, motor, cognitive, language, and social skills [1]. While many children enjoy healthy developmental trajectories, others falter because of mental, social, and behavioral health challenges that impact their development and chances of succeeding socially, academically, and financially [2]. Furthermore, developmental delays compromise children’s ability to function as productive community members. Prior evidence has highlighted the risk of millions of children in low- and middle-income countries (LMICs) failing to attain full cognitive development (e.g., [3]). Nigeria, the focus of this study, has over 6 million children under five years who are at risk of not reaching their developmental milestones [4,5,6].

Further, scholars note the paucity of culturally and context-based early childhood development (ECD) assessment instruments for tracking progress in LMICs [7]. The misalignment between assessment instruments and the context may lead to biased and unreliable data. For instance, Nigeria’s diverse language, education, socioeconomic, and cultural nuances also pose a challenge to the direct application of ECD instruments commonly used in high-income country contexts. Remediating this gap in culturally appropriate instruments calls for assessment tools that are well aligned with the culture and the context in which the children and their families are located. A focus on assessment instruments is important in ensuring accountability and responsive programming and interventions for the children served. Entities involved in ECD monitor, diagnose, and strengthen school programs and plan early interventions for children. Additionally, in therapeutic settings, such preliminary child development assessments are conducted to identify and manage progress in children’s developmental stages. Families, caregivers, or professionals are often asked to complete screening and assessment instruments focused on specific or multiple ECD domains [8,9]. Some measures are diagnostic in nature while other population-based measures provide a snapshot of whether the child is on track.

The Early Childhood Development Index (ECDI) has been used in the Multiple Indicator Cluster Surveys (MICS) and in over 110 countries to track ECD. We examine its use to track development among children aged 3 to 4 years in Nigeria in the 2016–2017 MICS. The evidence in place suggests that the ECDI was validated for use in Kenya [10]. To our knowledge, limited research has reported the utility and psychometric performance of the ECDI in other African countries including Nigeria. Understanding the performance of the ECDI in this context is useful even at the launch of the new ECD2030 [11]. Findings from this study have the potential to highlight areas for engagement and development of context-responsive multi-sectoral and child-centered interventions. Moreover, the findings may provide a framework for engaging the new ECD2030 in a reflective dialogue on the need for a measure that is aligned with the context, culture, and values of the children served.

### Review of the Literature

Monitoring Early Childhood Milestones.

Child development spans the evolution of a child to a teenager “in all areas of human functioning—social and emotional, cognitive, communication and movement” [12]. Some scholars define ECD as the period from zero to eight years of age whereas others utilize an age limit of five years [12]. Life course perspective scholars define childhood as the prenatal period (from conception to birth), infancy as the first 2 years of life, early childhood as 3–5 years, and middle childhood as the ages of 6–11 [13]. The literature contends that a child’s brain undergoes rapid growth from conception to approximately 36 months. The child’s unique environment, influenced by factors such as nutrition, stimulations, and positive or negative stressors, interacts to affect further development in cognitive and social skills [14]. The physical/motor, language, mental, emotional, and social dimensions constitute the most common and interdependent domains examined in ECD studies [12].

The physical domain consists of internal and external organs, as well as motor and cognitive abilities. As children develop physically, they attain movement and increased perception of their environment. By age two, a child’s brain attains 75% of its adult weight. The mental domain of ECD encompasses the five senses, and the mind–body coordination facilitated by the expansion of the brain and neural connections. The language domain represents the child’s ability to comprehend and use language to communicate their wants and needs. Although there is variation in the development of verbal communication skills, most children can form simple sentences by age two. The emotional domain sets the foundation for children to experience, interpret, and express “attachment, trust, security, love, and affection” that influence stability [15]. The social domain involves interacting with others, learning to build relationships, and reading the social cues of others. Both domains are usually examined together as a child’s socioemotional development.

As observed across cultures, several milestones mark the developmental progress of children. Although the WHO Guide for Monitoring Child Development (GMCD) posits that many ECD markers are similar across country and cultural contexts and that there are commonalities in the age at which children in these diverse contexts achieve overall motor milestones [12,16], it is still unclear whether the developmental trajectory of all well-children in different social and cultural contexts is comparable [17]. Furthermore, there is still a lack of consensus about what the ideal milestone for each developmental stage is. Family and community expectations for normal child development may also differ across geographies, highlighting the need for culturally appropriate assessment tools.

Furthermore, given the impact of biological and/or socioeconomic factors on child development, there is a need for indicators that effectively capture the local reality in assessing whether a child is on track developmentally. Monitoring and measuring child development has been described as a concern for child and human rights, demanding accountability from all duty bearers, including the government and primary caregivers [10,18]. ECD was included for the first time in the Sustainable Development Goals (SDGs), specifically under goal #4: ensure inclusive and equitable quality education. This signifies the international community’s endorsement of ECD as an essential component of child development globally [19,20]. When the developmental trajectory of the child is regularly and accurately monitored, risk factors associated with failure to attain milestones may be identified on time, and early interventions initiated [12,20]. However, many LMICs do not have mechanisms to monitor ECD trajectories for research, screening, or diagnostic purposes [21], where these exist, and assessment is limited to the use of proxy measures [17]. The scarcity of contextually relevant measures of ECD in LMICs may lead to biased estimations, poor planning, and misallocation of resources, undermining the development of responsive interventions.

To contribute to the discussion on the need for culturally and contextually sensitive and relevant measures for monitoring ECD, this study assessed the utility of the 10-item ECDI used in the MICS [22] to track developmental milestones among children under the age of five. We specifically focus on Nigeria, a country with an estimated population of over 200 million. Nigeria experiences heightened levels of poverty [23], high levels of daily life stressors, armed conflict, and high numbers of children at risk of failing to attain their full developmental potential [24,25,26,27]. Indeed, this context provides an opportunity for a contextualized exploration of the utility of ECD measures at the population level in Nigeria.

The Early Childhood Development Index (ECDI).

As mentioned above, the United Nations and expert partners developed the ECDI to support the SDGs that were designed to ensure equitable quality education and life-long learning opportunities [10]. This measure accounted for cost, time constraints, and ease of access. Over time, the ECDI has evolved from an initial 48-item version to the 18-item, and the 10-item version, which is utilized in this study. Based on the ECDI, a child aged 3 to 4 years old is on track overall if they are making progress in three out of the four domains: literacy–numeracy, approaches to learning, physical, and socio-emotional. Furthermore, when computed separately, aggregated results for each domain, along with the total ECDI count [10], constitute children on track in at least three of the four domains. Focusing specifically on Nigerian children, this study examined the utility of the 10-item version of the ECDI instrument (see Table 1), which has been assessed in Kenya, Jordan, and the Philippines, and used in more than 300 surveys, and over six rounds globally [10,28].

Definitions and Theoretical Framework for Validity and Reliability Assessment. This study employs two theoretical frameworks, namely the classical test theory (CTT) and the item response theory (IRT), to assess the validity and reliability of the ECDI. The CTT posits that a true score on any test is unobserved and theoretical. It is not directly related to the construct in question [29] but represents an individual’s expected value. The expected value of an individual can be influenced by knowledge, distractions, state of health, and mistakes, as well as guessing [30]. The CTT focuses on three types of test score reliability of the overall scale, namely internal consistency, test–retest, and parallel form. Per the CTT, a child’s observed score on the ECDI equals the true score plus random measurement error (x = Tx + e) [29]. Investigating the ECDI solely based on the face value of the CTT assumes that caregivers responding on children’s behalf attribute their development levels to reflect their actual ability in that domain [31]. Additionally, the CTT assumes that all the scores provided are equally valid, implying that the ECDI is accurate across all children, irrespective of their ability, and that different domains of development are interchangeable. Relying entirely on the CTT to determine the ECDI’s success in estimating ECD has drawbacks including difficulties in differentiating items with common themes and an overreliance on the sample of individuals [32]. In addition to the CTT, this study employs the one-parameter logistic model of the IRT to examine and provide a robust assessment of the ECDI items.

The item response theory (IRT) scores trait proficiency by highlighting the relationship between the trait and the response provided to it. Consequently, the IRT examines the probability of a person “succeeding” on a given item or test question [33,34]. The IRT is a probability model that technically measures the likelihood of different responses to an item. Unlike the CTT, which assumes that everyone responds to similar items similarly, the IRT considers the challenges in answering the question the item is measuring, as well as the discrimination, reliability, precision, or reproducibility of the score. A more discriminating instrument is more reliable when it consistently provides the same estimate. The IRT therefore tests the degree of endorsement of each response or the pattern and strength of endorsement of each response compared to the underlying construct [34,35].

The IRT makes assumptions of monotonicity, uni-dimensionality, item variance, and local independence [34,36]. Monotonicity assumes that the trait, such as a child’s ECDI, increases or the likelihood of a positive response to an ECDI question increases as the ability increases. This concept is best described on the IRT’s visual item characteristic curve (ICC). Uni-dimensionality assumes that there is only one dominant latent trait being measured, which acts as the driving force for the observed response and is confirmed by confirmatory factor analysis (CFA) fit statistics [36]. The invariance assumption of the IRT posits that the ability or ECD level is independent of the characteristics of the sample across subpopulations [34], like gender, age, and ethnicity, and is examined using the differential item functioning (DIF) statistic. The local independence assumption holds that responses provided by participants (in this case, mothers for their children) are not statistically associated with each other, although this may be sometimes violated in negatively worded items [34].

The IRT aims to differentiate among the levels of item response in the construct [37]. The IRT’s ICC or ICF is a sigmoid line that describes the probability of a person saying yes or succeeding on a given item. For example, concerning the ECDI, the IRT question will be “How likely are mothers to provide a “yes” to questions about the level of their child’s development in any given ECD domain?” This study assumes that mothers will have a positive response to an item depending on how much of the trait they believe their child has and how difficult or intense the item is [34]. Applying the IRT as a probability model in this study ensures that the underlying ECDI domain in each child is captured independently of the items used to assess its level, even though it is latent. Therefore, the performance of every child in an ECDI domain (not their true scores as assigned by the mother) will be a joint function of their overall ability (termed theta) and ECDI test item features. The IRT standardizes the scores from each ECDI item into the same metric to customize each child’s item, providing a precision index for the ECDI scale—a standard error measurement for each child [34]. Further, each child’s ECDI is based on the difficulty level and discrimination of the item and estimated through the likelihood of different responses by different levels of ECD.

Item difficulty. Since the item response for the ECDI is dichotomous (yes/no), the IRT equation for the question, ‘What is the probability, P of responding “yes” for the trait “theta θ” to an item i (that is (Pi = yes/θ), (where θ = the level of ECDI domain or trait of a person n responding to item i, error, e = 2.7 a fixed value, and β = beta is the difficulty of the item) is

Pi = yes/θ = e (θn − β1)

1 + e (θn − β1)

As shown above, this probability of a yes depends on how difficult the item is and on the value of theta (amount of trait). On the ICC (ogive or sigmoid curve) generated by an IRT analysis, the prediction line predicts the probability for every level of the trait measured. Also, on the continuum of the prediction line, 0.5 probability of a yes is the point of median probability—where there is a 50:50 chance of saying yes, which has different logits for each item. At lower logits, the item is easier to estimate. The slope of the prediction line is the item’s difficulty. Item difficulty, denoted by β, is the likelihood that each mother will respond correctly to the ECDI question (not in terms of the perceived difficulty or amount of effort required). In highlighting item difficulty, the IRT shows how sensitive the ECDI is, to capture both the strongest and the weakest development levels.

Item discrimination. For different items on a scale, the slope can be equal in difficulty but different in discriminatory power at the point of median probability where the trait meets the slope. Discrimination, denoted by α, is the instrument’s ability to differentiate between children with low and high levels of a trait. A high discrimination suggests that the item or instrument has a high ability to differentiate between the levels of development of the children.

To compute the discriminatory level of a measure or slope, the probability P

Pi = yes/θ = eα(θn − β1)

1 + eα (θn − β1)     where the slope α = discrimination

The present study. Employing the CRT and IRT, this study examined the utility of the ECDI instrument by addressing the following hypotheses:

i. There is an underlying factorial structure of the ECDI items.
ii. The ECDI demonstrates acceptable power in distinguishing between children whose development is on track and those who are not.

## 2. Materials and Methods

### 2.1. Study Design and Sample

This study used data from the fifth round of the 2016–2017 MICS conducted in Nigeria [22]. The sampling frame of the MICS is the updated Nigeria 2015 population–housing census, with primary sampling units (PSUs) in both rural and urban areas. The PSUs are 60 census enumeration areas (EAs) within each of Nigeria’s 36 states where 16 households per EA, per state, were sampled. Within the child questionnaires, caregivers (mostly mothers) through face-to-face interviews responded to 10 questions that constitute the ECDI about their under-5 child. The total sample in this study consisted of 11,207 Nigerian children at the age of 3 and 4 years old.

### 2.2. Measures

The ECDI asked questions relating to the child’s ability for traits expected for their age. The 10 binary response questions (yes/no) covered 4 domains, namely literacy–numeracy (3 items), physical (2 items), cognitive development/approaches to learning (2 items), and socioemotional (3 items) (see Table 1). The child demographic variables, age (in years), gender (male or female), place of dwelling (rural or urban), maternal (level of education), and household characteristics (wealth index, religion), were also examined.

### 2.3. Analytical Approach

Using the Stata 16 E statistical package, the data were cleaned and examined for outliers. The three negative items in the physical and socioemotional domains were reverse-coded. To ascertain the reliability and properties of the ECDI, this study applied the classical test theory (CTT) [32] and the item response theory (IRT) [34]. The IRT modeling was also used to examine the differential functioning of each item and the degree to which the items measure the desired ECD construct specifically and/or if other underlying constructs are represented. Correlations among the items and chi-squared difference in means were also determined. The sample was randomly split to provide calibration of appropriate sizes to ensure the accuracy of item parameter estimates [38]. Cronbach’s alpha was used to determine the internal consistency reliability. The principal axis factoring (paf) method was used for exploratory factor analysis (EFA), and the maximum likelihood (ml) method highlighted the factor structure. The oblimin oblique rotation method was used in the assumption that latent factors were corrected with each other.

A CFA of the dimensionality of the ECDI domains, as a one- or four-factor model based on the literature [10], was conducted. For model fit, recommended tests such as the Root Mean Square Error of Approximation (RSMEA), comparative fit index (CFI), Tucker Lewis Index (TLI), and Standardized Root Mean Square Residual (SRMR) were used for comparison [39]. The Kuder–Richardson coefficient of reliability (KR20) computed the item difficulty (proportion of “right” answers), and the average value of item difficulty for dichotomous items and estimated the ECDI reliability across different random sets of items. The 1PL IRT model was estimated to highlight the utility of the instrument [40]. All assumptions such as independent sampling, normality, and linearity were largely met. Missing items were less than 0.03%.

## 3. Results

### 3.1. Demographic Statistics

As shown in Table 2, of the overall sample of 11,207 children (3 years old, 51%, and 49% female) from which calibration samples were taken, 74% resided in rural areas. Most responding mothers were between 30 and 39 years old (44%), followed by between 20 and 29 years old (39%), between 40 and 49 years old (15%), and between 15 and 19 years old (2%). Most (65%) had no education. Further, based on the ECDI, over 50% of the children’s milestones were on track.

### 3.2. Correlation

The ECDI items had a range of strong to weak correlations with each other that were statistically significant (*p* < 0.001) (see Table 3). Within domains, the three literacy–numeracy domain items were most correlated with each other (0.62, 0.76, 0.60). There was no linear relationship between the two socioemotional items (aggressive behavior and attention). The socioemotional items were the least correlated with all others. The strongest correlation was between physical development and cognitive/approaches to learning (r = 0.31). There was a negative correlation between socioemotional development, learning, and the physical domain (r = −0.23).

### 3.3. Internal Consistency

The Cronbach’s alpha of the 10 ECDI items was 0.65. At the domain level, the three literacy–numeracy items had an internal reliability (alpha α) of 0.85, the physical development domain items α = 0.10, the cognitive domain items α = 0.71, and the socioemotional development domain items, α = 0.36. Removing the physical domain item for sickness increases the alpha to 0.67 and when in the socioemotional item, attention is removed, the ECDI alpha increases to 0.75. The alpha of the 10 items across child demographic characteristics such as age was 0.68 when children are 3 years and increased to 0.71 for 4-year-olds. For children in the urban areas, α = 0.67, and for the rural areas, α = 0.69. By gender, α = 0.70 for males and 0.71 for females (see Table 4). Using Raykov’s reliability coefficient to further test the composite item reliability when they are congeneric and uni-dimensional, the ECDI had a value of 0.415 and 0.520 (when physical development items are removed). A value greater than or equal to 0.7 is usually preferred [41].

### 3.4. The Kuder–Richardson Coefficient of Reliability (KR-20)

For complementary estimates of reliability, the KR-20 estimate for dichotomous responses was used to further test the reliability of the ECDI and indicated how consistent the results were and how well the ECDI performed [30]. The KR-20 value for the ECDI items was 0.67. The KR-20 value for the ECDI for males was 0.67, for females 0.68, urban 0.67, and in rural areas, 0.64. The KR-20 value (0.67) indicates that the ECDI items are just acceptable or reasonable (where 0 is null reliability and 1 is perfect reliability). A higher value above 0.70 is usually preferred [30].

### 3.5. Measure of Sampling Adequacy (MSA)

The Kaiser–Meyer–Olin (KMO) test of an identity matrix (KMO value 0.74) indicated the sufficiency of the items for each factor, with two eigenvalues above one. The SMC value of each item with all other items ranged from 0.62 for literacy–numeracy to a weak 0.08 for the socioemotional items. A parallel analysis with predicted values yielded an estimate of the same factor model as the original, confirming the retained factors.

### 3.6. EFA Results

The principal axis factoring (paf) EFA and parallel analysis supported a four-factor model, as laid out by the ECDI designers [10]. As shown in Table 5, the proportion of variance accounted for by the first factor was 75%. Most items showed that less than 0.29 of the variances were unique to them. The physical (frequently sick) and socioemotional domains had high uniqueness values of variance not shared with others. Communalities (index of the expression of common variance) from the squared multiple correlations (smc) indicated that the numeracy–literacy domain items had the highest communalities (0.71, 0.49, and 0.70), meaning that this default model does the best job of explaining variation in these items/factor model. The physical items (being sick/grasping) had the lowest communality where only 16% of its variation was explained by this model, as with the socioemotional items (18%). The oblimin oblique rotation, where factors were correlated, clearly partitioned the proposed literacy–numeracy factor and cognitive items, two of the socioemotional items, leaving an ambiguous placement of the physical domain and one socioemotional domain item, also highlighted in a loading plot. However, the uniqueness values did not change, indicating that rotation did not affect the proportion of variance explained.

### 3.7. Uni-Dimensionality and CFA Results

The goodness-of-fit statistics from all the models suggest that the one-factor model, compared to the four-factor solution, did not fit the data well (chi-squared, *p* < 0.001). The modified final model of the four-factor solution yielded adequate fit and minor modifications, chi-squared 503.2, and more degrees of freedom, 29, (*p* < 0.001). The 90% confidence interval for the RMSEA was 0.067 (ideal range 0.05–0.08), and the comparative fit index (CFI) was 0.94 (recommended 0.90–0.95) [39,42].

As presented in Table 6, the SRMR value of 0.05 means that on average, the four-factor ECDI model comes within the 0.05 range of reproducing the correlations among the items (ideal value, less than 0.08 [39]. Other model fit indices AIC and BIC were lower for the four-factor model than the one-factor model. When the CFA for the final model is repeated by gender, age, and place of dwelling, the fit and factor loadings showed similar stability for a four-factor model.

### 3.8. IRT Modeling

The difficulty parameters (b) highlighted the underlying structure of the ECD through item location. A good instrument has item difficulties spread across the full range of the trait (thus differentiating the level of ECDI across all levels). The item characteristic curves (ICC) show the difficulty spectrum of all items, where the probabilities represent the expected scores for each ECDI variable along a continuum. The ICC was different for each ECDI item and as the difficulty level of an item increases, the curve shifts along the ability scale (*x*-axis), meeting the monotonicity assumption of the IRT, that the likelihood of a positive response increases with the ECD.

**Figure 1:**
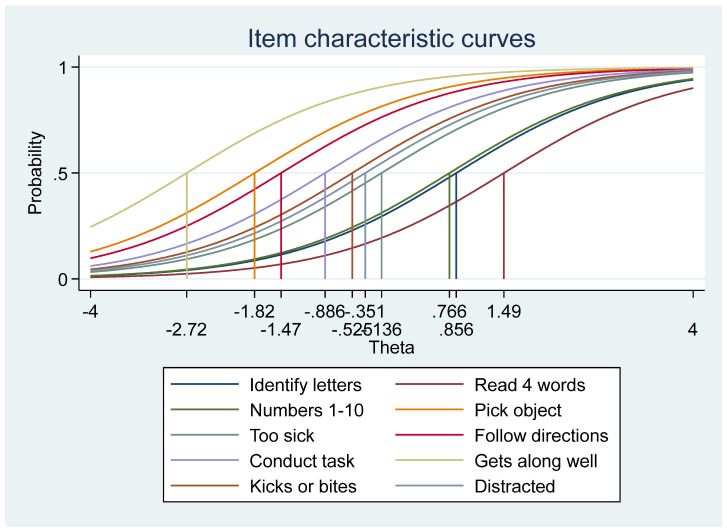
Item Characteristic Curves (ICC), showing the difficulty of each ECDI item. Notes: As shown in the graph legend, the different colored lines represent each ECDI item, and how they are arranged according to their difficulty spectrum, determined at the point of medium probability (0.5) on the ICC.

The item difficulty spectrum (Figure 1, Table 7), determined at the point of medium probability (0.5) on the ICC, ranged from the least difficult—the socioemotional item (child gets along with others) at −2.54 to the literacy–numeracy item (reading at least four-letter words) at 1.18, being the most difficult. The range is usually from −4 and +4. These values mean that the probability of success on the socioemotional item is higher than in the other traits, at any ability level, and that the literacy–numeracy item being more difficult and shifted right indicated the higher ability of positive endorsements.

Overall, the one-parameter logistic (1PL) model yielded an item discrimination parameter or slope (constrained for all items) of 0.99, suggesting that the items are not discriminating [33], such that any two children with distinct stages of development would have similar predicted probability of responding correctly to the question. The discrimination (slope α) highlighted how fast the probability of a yes response changes with the ECDI when the item is difficult. The curves illustrated precisely the items measured by the ECDI at every level of development (reliability) on a comparable standard scale. 

The Item Information Functions (IIFs) (how much information is contained in each item, relative to the data) of the items were unimodal and symmetric, indicating how each item provides the maximum amount of information at its estimated difficulty parameter where the height is proportional to its discrimination parameter. An overall test information function (TIF), where all curves were constrained to be of the same height or discrimination, showed the sample ECDI ease, indicating that most children are at Theta = 0, as few are at the very low or very high range. Based on the test information curve (TIF), the ECDI provided the maximum information for children approximately located at the ability level, theta (θ). From this ability level to either direction (increasing or lowered), the standard error increases, indicating that the information the instrument provided about a child’s ECDI is decreasing, and not robust. 

Furthermore, a test of the item fit for the 1PL model was conducted by superimposing empirical proportions of a sample item on the TCC—where if there is a close alignment of the predicted ICC with the empirical trace line as implied by the proportions, then the model fits or the items are assumed to have a satisfactory fit [33]. The invariance assumption of the IRT will hold true for the ECDI items only if the model fits the data. The overall Test Characteristic Curve (TCC), indicating the monotonicity of the ECDI instrument, and ranging from 0 –10 score. 

**Figure 2:**
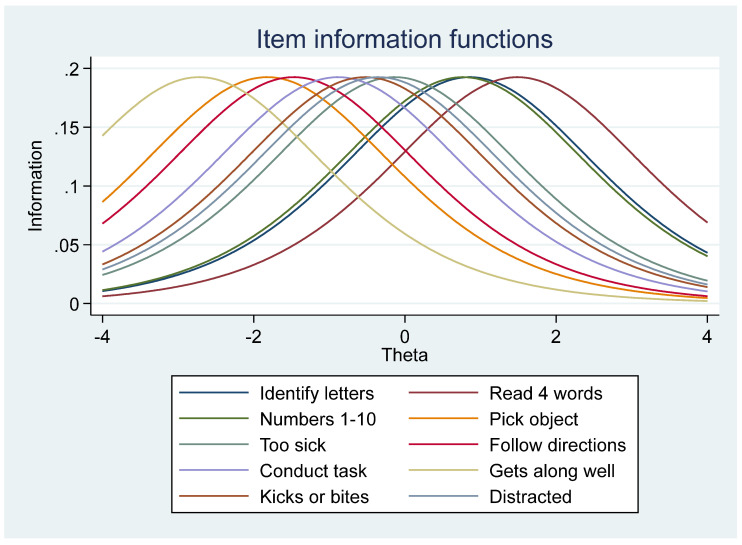
1PL model misfit (sample using the literacy–numeracy item).

A sample literacy–numeracy item ICC superimposed on the predicted value, see Figure 2, indicated a poor fit, the same for all ECDI items. Further, an examination of the differential item functioning (DIF) of the ECDI items along the demographic areas of a child’s household wealth, rural, and urban dwelling across groups did not yield converging results, thus violating the invariance assumption of the IRT.

## 4. Discussion

This study set out to examine the utility of the ECDI instrument in tracking the developmental milestones among 3- to 4-year-old children in Nigeria. The 10-item ECDI had a low to average internal consistency (Cronbach’s alpha = 0.65) in the sample, suggesting that some of the items in the ECDI may not be representative of the underlying construct or are too few, depending on the school of thought. At the domain level, only the literacy/numeracy and the cognitive domains had acceptable internal consistency (alpha 0.85 and 0.71, respectively). The physical and socioemotional domain items had very low Cronbach’s alphas (alpha 0.10 and 0.036, respectively), corroborating with past studies suggesting that these items may not be valid constituents of the ECDI [5,42,43], or that the stipulated level of trait per domain expected for these age groups within the instrument is too high.

**Figure 3:**
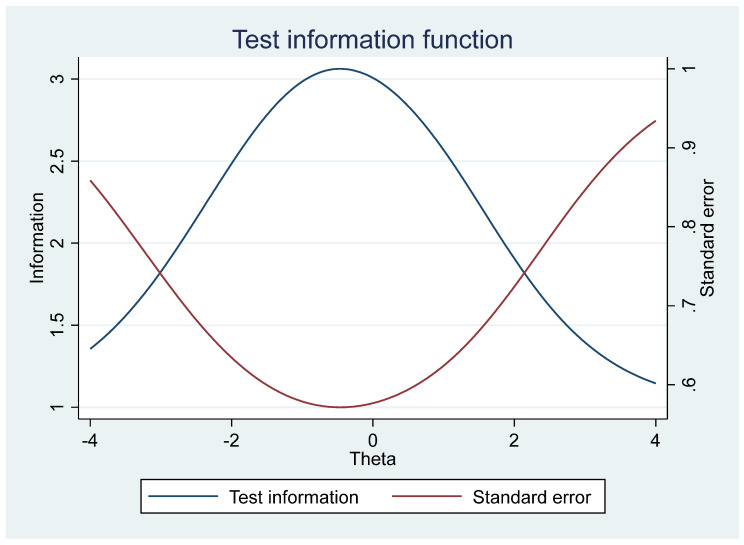
Illustration of standardized coefficients of a confirmatory factor analysis for the ECDI items.

Based on the literature, the ECDI’s physical domain measure—the pincer grasp—is too elementary for a healthy 3- to 4-year-old child and rather a milestone expected for a 9- to 12-month-old child [12]. More appropriate fine motor skills for a 3-year-old could include the ability to copy a circle or put on shoes while the ability to copy a square or use scissors would be a more appropriate measure for a 4-year-old [16], depending on contexts and socioeconomic factors. Further, the item asking if the child is frequently sick is more of an index of health status, as diseases, and not developmental delay, may be associated with frequent sickness. The data analysis reinforced the need to re-examine the physical domain items as the elimination of the physical domain item (frequent illness) increased the ECDI alpha to 0.67. Further, the physical domain low factor loadings also suggested concerns about their inclusion in the ECDI (see Figure 3).

The ECDI’s three items for the socioemotional development domain (Table 1) that asked questions relating to social competence, behavior, and attention problems are in line with previous research and milestones for this age [15]. However, in terms of face validity, the distraction question may pose a challenge in the Nigerian multicultural and multi-lingual (over 300 languages) milieu. Asking if a child is easily distracted may be misinterpreted by the mother and may account for its poor performance on the scale. Other factors such as hunger or poor diet, lack of sleep, or conflict in the home unrelated to child development may lead to distraction. Overall, the socioemotional items do not seem to capture any clear construct, nor load to one latent factor, thus reducing the ECDI’s validity and uni-dimensionality in measurement.

Based on the analysis, when the socioemotional attention item (which had a low inter-item, and internal consistency reliability (0.36), and the least correlations with other items in the ECDI domains) was removed, there was an improvement in the ECDI Cronbach’s alpha (0.70), inferring that the instrument could be optimized overall by dropping or replacing this item. Overall, the socioemotional development items exhibited a poor fit, low correlations, and the lowest communality of 18% in the EFA. The debated items in the ECDI presented problematic results in all the statistical analyses. Further, the EFA showed that the Cronbach’s alpha of the ECDI varied across sub-groups. For instance, across educational levels, the ECDI items had an alpha of 0.60 for responding mothers with higher education, 0.63 (for mothers with secondary and secondary technical education), 0.63 (for mothers with primary education), and 0.69 for mothers with non-formal education. Across ethnicities, for mothers who identified as Hausa, the internal consistency of the items was 0.67, Yoruba 0.67, Igbo 0.68, and all other ethnicities combined 0.65. Elimination of the socioemotional items (for distraction/attention) for these groups saw an increase in the internal consistency of the ECDI to 0.75, which should be a minimum for use in any setting.

As observed by other scholars [5], the ECDI items measuring literacy and numeracy may be too advanced for children 3 to 4 years of age in LMIC settings. Indeed, given the lower levels of maternal education and high levels of poverty in many Nigerian communities, it may be harder for households to have resources to facilitate a higher level of literacy and numeracy skills development [43]. However, the response from the caregivers showed consistently low percentages of children who have attained the literacy–numeracy milestone (mostly the 4-year-old children). The mothers’ acknowledgment of their child’s inability to perform the domain-related tasks gives credibility to the data in the sense that there is a low sense of social desirability. The analysis supports that the literacy/numeracy measures are valid and have high internal reliability and consistency overall. The literacy–numeracy items are also consistently loaded to one factor, showing uni-dimensionality.

Overall, the EFA findings suggest that the dimensionality of the ECDI items among the Nigerian children suited a four-factor, rather than a one-factor, ECDI structure/interpretation in defining overall progress in development. The poor fit of the one-factor model as opposed to the four-factor model violates the IRT assumption of uni-dimensionality, that there is only one dominant latent trait being measured and that is the driving force for the response observed. The IRT modeling further helped to unpack the ECDI items and highlighted individual item precision at different levels of difficulty. The overall Test Characteristic Curve (TCC) showed that the ECDI construct fulfilled the IRT requirement of monotonicity, where the probability of a positive response increased with ability. As depicted in the IPL IRT model estimates, the physical and socioemotional domain items within the construct had the lowest difficulty parameters while the literacy–numeracy items had the highest difficulty estimates.

Based on the 1PL model, the test information function (TIF) curves were the same height for each ECDI item and clustered in the middle, confirming that the ECDI covers a wide range of different levels of child development and at moderate to low levels of traits [33]. The individual TIF curves of the items also had several overlaps, calling for a review of the items. The convergence of the 2PL model was challenging, leaving the discrimination for only one item constrained. Scholars suggest that the lack of convergence may be an indication of poor model fit arising from “too many poorly fitting observations” [33,44], thus supporting previous findings that the scale may be problematic.

The poor statistical outcomes of the measure and the physical domain construct may mean that the instrument is not adequately capturing information of value to support an understanding of the development of children in the Nigerian sample. Yet, the important information on frequent sickness may be a factor in a child not achieving other milestones and draw attention to the need to improve access to healthcare services. To the credit of the children and their families, most (over 90%) were on track in the socioemotional development items. This observation likely reflects the role Nigeria’s collectivist culture may play in mitigating social-emotional wellbeing. We noted that the literacy/numeracy items were higher in their difficulty level and discriminatory power. In line with previous research [43], only 42% of the children were on track in this domain. This result underscores the need for re-examination of the numeracy items within this context. Overall, there was little variation in the performance of ECDI items by ethnicity. However, variations were observed by household wealth and dwelling (rural/urban). This may be explained by their association with family socioeconomic characteristics [43,45,46]. Overall, the family environment can play a key role in child development [45,47]. It is also important to consider a multi-dimensional approach, encompassing community, as well as family, for child development.

Study Limitations. As reported in similar studies [43], parental responses to the ECDI may be affected by recall bias, social desirability, and maternal personality type. In addition, we used a cross-sectional dataset; this limited our ability to analyze other aspects of instrument validity. While our study used several robust statistical procedures to review the ECDI, there are chances that alternative procedures not utilized may shed more light on the instrument. We also acknowledge that the lack of a benchmark to compare the ECDI precluded the confirmation of its postdictive or convergent validity. Although this study is not based on the recently launched ECDI2030 [48], it has utility in that policies and intervention decisions continue to be made based on the ECDI tracking. This necessitates continued dialogue on early childhood developmental trajectories guided by ECDI data. Further, this study would have benefited from a comparative analysis of the ECDI’s performance in other African countries; however, it still has important practice implications including serving as a benchmark for future research using the ECDI2030.

## 5. Conclusions

This study examined the utility of the ECDI used among children between the ages of 3 to 4 in Nigeria. This study acknowledges that the ECDI has had positive transcultural epidemiology having been used successfully in over 110 countries [49]. Indeed, the questions asked in the ECDI are useful for child studies and provide a useful way to examine different aspects of the cognitive and social development of children. The results of the current study suggest that a four-factor model of the ECDI had a better fit than a one-factor model, and so may provide utility for assessing specific domains of development rather than interpreting ECD overall. None of the fit indices such as the RMSEA, CFI, and TLI values indicated a good fit (less than 0.90). Further, the weak correlations and the low discriminatory power of some items point to the necessity to review the criterion validity of the ECDI.

At the policy level, tracking children’s developmental progress can be instrumental in monitoring trends in prevalence across populations, increasing knowledge about demographic and geographic differences, and supporting decision-making about prevention and intervention [43,47,50]. Furthermore, when communities participate in surveys examining ECD, externalities such as increased awareness, capacity building, and community participation accrue. Beyond policymakers (who are charged with resource allocation) and practitioners who intervene, questions about ECD measures asked in the populace help to orient caregivers to expectations for ECD. Another implication of this study is the need for a discourse bringing together multiple stakeholders including families, practitioners, and policymakers to review shortcomings, if any, in ECD instruments and adjust interventions informed by this measure. Indeed, increased discourse is essential for the development of culturally appropriate and child-centered measures on child development that are well aligned with the context, culture, and values of the children and families served [47].

A revised cognitive testing focused on the ECDI2030, comprising 3 domains (learning, health, and psychosocial wellbeing) and 12 subdomains that include literacy, numeracy, executive functioning, self-care, gross and fine motor, expressive language, pre-writing, emotional and social skills, and mental health across 20 items, has been launched [11,48]. Continued dialogue on ECD measures that reflect the lived experiences of children served is important, especially as countries begin to extensively use the newly launched ECDI2030. Research efforts could be devoted to assessing the utility and psychometric performance of the ECDI2023 in LMICs, an ethical and professional responsibility.

## Figures and Tables

**Table 1:** The 10-item ECDI measure.

10-Item ECDI	
Literacy–numeracy	EC8. Can (name) identify or name at least ten letters of the alphabet?
	EC9. Can (name) read at least four simple, popular words?
	EC10. Does (name) know the name and recognize the symbol of all numbers from 1 to 10 most of the time?
Physical	EC11. Can (name) pick up a small object with two fingers, like a stick or a rock, from the ground?
	EC12. Is (name) sometimes too sick to play?
Approaches to learning	EC13. Does (name) follow the simple directions on how to do something correctly?
	EC14. When given something to do, is (name) able to do it independently?
Socioemotional	EC15. Does (name) get along well with other children?
	EC16. Does (name) kick, bite, or hit other children or adults?
	EC17. Does (name) get distracted easily?

**Table 2:** Demographic characteristics of children reported in the ECDI indicators (n = 11,073).

Characteristic	n (%)
Child’s Age	
3 years	5643 (51)
4 years	5430 (49)
Child’s Gender	
Male	5632 (51)
Female	5441 (49)
Household Dwelling	
Rural	8140 (74)
Urban	2933 (26)
Mother’s Age	
15–19 years	156 (2)
20–29 years	4016 (39)
30–39 years	4425 (44)
40–49 years	1556 (15)
Mother’s Educational Level	
None	3141 (28)
Primary/Non-formal	4123 (37)
Secondary/Higher	3808 (34)

**Table 3:** Correlation of ECDI items among 3- to 4-year-old Nigerian children (n = 11,073).

		ECDI Items	1	2	3	4	5	6	7	8	9	10
Literacy–numeracy	1	Identify letters	1									
	2	Read four words	0.62	1								
	3	Numbers 1 to 10	0.76	0.60	1							
Physical	4	Pick object	0.20	0.18	0.21	1						
	5	Too sick ^c^	0.03	0.02	0.06	−0.11	1					
Cognitive–approaches	6	Follow direction	0.21	0.18	0.21	0.31	−0.07	1				
	7	Conducts a task	0.18	0.16	0.19	0.25	−0.04	0.56	1			
Socioemotional	8	Gets along well	0.14	0.12	0.15	0.32	−0.12	0.34	0.27	1		
	9	Kicks/bites ^c^	0.00	0.01	0.00	−0.09	0.19	−0.07	−0.01	−0.10	1	
	10	Distracted ^c^	0.02	0.01	0.01	−0.02	0.20	−0.04	0.01	−0.11	0.25	1

Note. All correlations were statistically significant (*p*< 0.001) except for the socioemotional item (9) and the literacy and numeracy items (1, 2, 3); and the socioemotional item (10) and the literacy and numeracy items (1, 2, 3), physical (4), and cognitive (7) items. Physical item (5) was mostly correlated with all socio-emotional items (8, 9, 10). Cognitive item (6) correlated with all items except too sick (5) ^c^ Denotes reverse-coded items.

**Table 4:** Item reliability, uniqueness, communality, and coefficients for ECDI items.

ECDI Items	Mean (SD)	KMO	SMC	COM	Overall Alpha α	Age 3 α	Age 4 α	Urban α	Rural α	Yes Edu α	No Edu α	Igbo α	Lang Yoruba α	Hausa α
Can identify letters	0.34 (0.47)	0.70	0.64	0.72	0.58	0.56	0.58	0.55	0.59	0.50	0.58	0.58	0.57	0.61
Read four words	0.24 (0.43)	0.84	0.45	0.51	0.60	0.59	0.60	0.59	0.61	0.55	0.59	0.61	0.61	0.63
Numbers 1 to 10	0.36 (0.48)	0.70	0.63	0.71	0.58	0.57	0.58	0.54	0.59	0.50	0.58	0.59	0.56	0.62
Pick small object	0.80 (0.40)	0.83	0.16	0.23	0.66	0.64	0.66	0.66	0.65	0.61	0.59	0.65	0.65	0.64
Too sick (re-coded)	0.47 (0.50)	0.66	0.09	0.17	0.69	0.67	0.70	0.68	0.68	0.63	0.62	0.69	0.68	0.67
Follows directions	0.75 (0.43)	0.68	0.38	0.50	0.65	0.63	0.66	0.65	0.64	0.60	0.58	0.65	0.65	0.63
Conducts a task	0.66 (0.47)	0.67	0.34	0.44	0.66	0.63	0.67	0.65	0.65	0.60	0.59	0.66	0.64	0.64
Gets along well	0.89 (0.31)	0.79	0.17	0.25	0.66	0.64	0.67	0.67	0.66	0.62	0.59	0.67	0.67	0.65
Kicks (re-coded)	0.60 (0.49)	0.63	0.10	0.19	0.69	0.67	0.69	0.68	0.66	0.62	0.62	0.69	0.68	0.67
Distracted (re-coded)	0.56 (0.50)	0.58	0.10	0.20	0.69	0.67	0.69	0.67	0.68	0.61	0.62	0.70	0.66	0.66
Overall	4.6 (0.40)	0.72			0.67	0.66	0.68	0.67	0.67	0.62	0.62	0.68	0.67	0.67

Notes: Yes/no Edu is whether the focal child attends an early education program or not, while language (Lang) represents the mother’s language.

**Table 5:** Exploratory factor analysis of ECDI items, oblimin oblique rotation (calibration sample, n = 3691).

ECDI Domains	ECDI Items	KMO	SMC	COM	Principal Axis Factors (paf)			
Factor					Factor 1	Factor 2	Factor 3	Factor 4
Literacy–Numeracy	Can identify letters	0.70	0.64	0.72	0.85			
	Read four words	0.84	0.45	0.51	0.71			
	Numbers 1 to 10	0.70	0.63	0.71	0.84			
Physical	Pick small object	0.83	0.16	0.23			0.44	
	Too sick ^c^	0.66	0.09	0.17				0.34
Cognitive–Learning	Follows directions	0.68	0.38	0.50		0.64		
	Conduct a task	0.67	0.34	0.44		0.69		
Socioemotional	Gets along well	0.79	0.17	0.25			0.41	
	Kicks or bites ^c^	0.63	0.10	0.19				0.44
	Distracted ^c^	0.58	0.10	0.20				0.45
	Overall	0.72						

Note: ^c^ Denotes reverse-coded items. KMO—Kaiser–Meyer–Olkin measure of sampling adequacy, SMC—squared multiple correlations of the variable with all other items, com—communalities, or index of expression of common variance.

**Table 6:** CFA estimates and fit indices for modified one-factor and four-factor model of ECDI items (calibration sample, n = 3690).

	Factor Loadings	One-Factor ECDI Model		Four-Factor ECDI Model	
	(Observed -> Latent Construct) Measurement Model	Unstandardized Coefficients (SE)	Standardized Coefficients (SE)	Unstandardized Coefficients (SE)	Standardized Coefficients (SE)
Literacy– Numeracy	Can identify letters	1 ***	0.87 (0.01) ***	1 ***	0.88(0.01) ***
	Read four words	0.69 (0.02) ***	0.68 (0.01) ***	0.69 (0.02) ***	0.68 (0.01) ***
	Numbers 1 to 10	1.0 (0.02) ***	0.87 (0.01) ***	1.0 (0.02) ***	0.88 (0.01) ***
Physical	Pick small object	0.26 (0.02) ***	0.27 (0.020) ***	1 ***	0.67 (0.06) ***
	Too sick (not)	0.04 (0.02)	0.03 (0.02)	−0.34 (0.05) ***	−0.18 (0.02) ***
Cognitive– Approach	Follows directions	0.29 (0.02) ***	0.30 (0.02) ***	1 ***	0.84 (0.02) ***
	Conducts a task	0.27 (0.02) ***	0.26 (0.02) ***	0.85 (0.04) ***	0.64 (0.06) ***
Socioemotional	Gets along well	0.13 (0.01) ***	0.18 (0.02) ***	1 ***	0.63 (0.03) ***
	Kicks or bites (not)	−0.02 (0.02)	−0.02 (0.02)	−0.54 (0.07) ***	−0.21 (0.02) ***
	Distracted (not)	0.00 (0.02)	0.00 (0.02)	−0.41 (0.06) ***	−0.16 (0.02) ***
Measurement Error Variances					
	Can identify letters	0.05 (0.00)	0.25 (0.01)	0.05 (0.00)	0.23 (0.01)
	Read four words	0.10 (0.00)	0.54 (0.01)	0.10 (0.00)	0.54 (0.01)
	Numbers 1 to 10	0.06 (0.00)	0.25 (0.01)	0.06 (0.00)	0.25 (0.01)
	Pick small object	0.15 (0.00)	0.93 (0.01)	0.09 (0.01)	0.55 (0.07)
	Too sick (not)	0.25 (0.01)	1.0 (0.00)	0.24 (0.01)	0.97 (0.01)
	Follows directions	0.17 (0.00)	0.91 (0.01)	0.06 (0.00)	0.30 (0.03)
	Conducts a task	0.21 (0.00)	0.93 (0.01)	0.13 (0.00)	0.59 (0.02)
	Gets along well	0.09 (0.00)	0.97 (0.01)	0.06 (0.00)	0.60 (0.04)
	Kicks or bites (not)	0.24 (0.01)	1.0 (0.00)	0.23 (0.01)	0.96 (0.01)
	Distracted (not)	0.25 (0.01)	1.0 (0.00)	0.24 (0.01)	0.98 (0.01)
R-Squared (Overall parameter fit)					
	Can identify letters		0.76		0.77
	Read four words		0.46		0.68
	Numbers 1 to 10		0.75		0.75
	Pick small object		0.07		0.44
	Too sick (not)		0.00		0.03
	Follows directions		0.08		0.70
	Conducts a task		0.06		0.41
	Gets along well		0.03		0.40
	Kicks or bites (not)		0.00		0.04
	Distracted (not)		0.00		0.02
	Overall		0.88		0.99
Fit Statistics					
	Chi-squared (df)		1600.28 (34) ***		503.25 (29) ***
	RMSEA		0.10		0.067
	CFI		0.82		0.94
	TLI		0.76		0.91
	SRMR		0.10		0.05
	CD		0.88		0.99
	AIC		37,669.56		36,582.54
	BIC		37,863.18		36,806.23

Note: *p* < 0.001 ***.

**Table 7:** Estimates from a 1PL IRT model of ECDI items among 3- to 4-year-old children (n = 581).

		1PL IRT Model Estimates		
Items	Item Definition	Yes (%)	Mean (SD) (Listwise)	Difficulty (SE)
	1PL overall item discrimination 0.99 (0.05) ***			
Can identify letters	Can (name) identify or name at least ten letters of the alphabet?	34	0.36 (0.48)	0.69 (0.11) ***
Read four words	Can (name) read at least four simple, popular words?	24	0.27 (0.45)	1.18 (0.12) ***
Numbers 1 to 10	Does (name) know the name and recognize the symbol of all numbers from 1 to 10 most of the time?	37	0.39 (0.49)	0.54 (0.11) ***
Pick small object	Can (name) pick up a small object with two fingers, like a stick or a rock, from the ground?	80	0.81 (0.40)	−1.71 (0.14) ***
Too sick	Is (name) sometimes too sick to play? (Re-coded)	47	0.51 (50)	−0.04 (0.10) ***
Follows directions ^c^	Does (name) follow the simple directions on how to do something correctly?	75	0.75 (0.43)	−1.34 (0.13) ***
Conducts a task	When given something to do, is (name) able to do it independently?	67	0.65 (0.48)	−0.75 (0.11) ***
Gets along well	Does (name) get along well with other children?	89	0.89 (0.30)	−2.54 (0.19) ***
Kicks or bites ^c^	Does (name) kick, bite, or hit other children or adults? (Re-coded)	60	0.43 (0.50)	0.37 (0.10) ***
Distracted ^c^	Does (name) get distracted easily? (Re-coded)	57	0.45 (0.50)	0.24 (0.10) ***

Note: All coefficients were statistically significant at the same value of * = *p* < 0.001 ***. ^c^ Denotes reverse-coded items.

## Data Availability

The data are de-identified and in the public domain at https://mics.unicef.org/surveys.
